# Dermatology in a British General Hospital

**Published:** 1918-10

**Authors:** 


### DERMATOLOGY AND UROLOGY

***Dermatology in a British General Hospital in France: Including the Differentiation of " I. C. T."*** By Captain FRANK CROZER KNOWLES, M. R. C., U. S. A. Dermatologist, U. S. A. Base Hospital, N° 10. Preliminary note of an article to appear elsewhere.

An account of the dermatological phase of the work of this hospital during the year of its direction by the American Army may prove interesting.

The staff of the Dermatological Department consists of the officer in charge, a non-commissioned officer, and two, occasionally three, enlisted men, while accommodation is provided for one hundred and twenty-eight cases.

The 1,913 cases which have been treated during this period of twelve months do not include patients who remained here for a few days en route for venereal hospitals, any cases of urticaria and toxic erythema which developed subsequent to admission, nor yet the very numerous battle casualties showing pediculi in their clothing, or " nits " on the pubic or axillary hairs.

The following table gives the variety and number of cases of each skin condition treated:

| | | | |
|---|---|---|---|
| Abscess | 50 | Hyperidrosis | 56 |
| Angioneurotic edema | 3 | Ichthyosis | 4 |
| Acne | 5 | Impetigo | 122 |
| Acne Varioliformis | 2 | Keratosis palmaris et plantaris | 1 |
| Alopecia Areata | 3 | Keloid | 1 |
| Boils | 381 | Lupus Erythematosus | 1 |
| Callositas | 8 | Lupus Vulgaris | 1 |
| Carbuncle | 7 | Molluscum contagiosum | 1 |
| Dermatitis herpetiformis | 1 | Pityriasis Rosea | 10 |
| Dermatitis | 35 | Psoriasis | 26 |
| Epithelioma | 2 | Pompholyx | 2 |
| Erythema Multiform | 4 | Papilloma | 2 |
| Erythema modosum | 1 | Purpura | 5 |
| Erythema (toxic) | 7 | Pediculosis Corporis | 10 |
| Eczema | 51 | Pediculosis Pubis | 8 |
| Eczema seborrhoeicum | 6 | Pernio (Frost-bite) | 50 |
| Ecthyma | 209 | Paronychia | 3 |
| Erysipeloid | 1 | Seborrhea | 5 |
| Folliculitis | 5 | Sycosis Vulgaris | 4 |
| Herpes simplex | 1 | Scabies | 510 |
| Herpes Zoster | 18 | | |

| | | | |
|---|---|---|---|
| Septic Ulcer . . . . . . . | 210 | Tinea Versicolor . . . . . | 3 |
| Schoelein's Disease . . . . | 1 | Tinea Sycosis. . . . . . . | 5 |
| Syphilis (secondarias) . . . | 37 | Urticaria. . . . . . . . . | 7 |
| Syphilis (late). . . . . . . | 15 | Vitiligo. . . . . . . . . . | 2 |
| Tinea Circinata. . . . . . | 2 | Von Recklinghausen's Disease . . . . . . . . . . | 1 |
| Tinea Cruris. . . . . . . | 6 | | |

In consideration of the vast numbers of sick and wounded to be dealt with, certain class names are given to various diseases, their correct differentiation remaining for later elucidation on the part of the specialist in the line to which the name is applicable.

One of the most interesting current class terms to the American dermatologist is " I. C. T. " (inflammation connective tissue), the synonym of the American Army term " Pyodermia ". These so-called " I. C. T. " cases are even eventually weeded out into their proper nomenclature, in the event that a trained dermatologist is present at their final destination. Like so many class terms, however, I. C. T. is frequently used to cover a diagnosis so diametrically opposite from the original intention that a dermatologist's opinion is absolutely necessary.

Out of the 1913 skin cases admitted to this hospital during the last twelve months, 995 have borne the diagnosis of I. C. T.

Several hundred other cases diagnosed as I. C. T. on the " field card " proved to comprise purely surgical conditions such as gangrene of the feet, advanced stages of so-called " Trench-Feet ", bone felon, necrosis of the bone, deep seated carbuncles, abcesses and flesh wounds of various types.

An important point requiring emphasis is that many skin conditions may prove unbenefited by treatment for a considerable period because of improper medication or because of an incorrect diagnosis. This latter point is well exemplified, as twenty cases of syphilis, eight in the second stage and twelve showing late skin lesions, were admitted to the hospitals improperly diagnosed.

Scabies also, under war conditions, differs greatly from that seen in civil practice, as the hands, and not infrequently the wrists, show little or no eruption, and pus complications are extremely frequent. Ecthyma, contrary to experience in civil practice, is here very prevalent as is also Septic Ulcer.

Emphasis should be laid upon the fact that greater care is required in diagnosing between active and non-active lesions of the skin. In numerous instances, cases have been sent to the hospital showing only pigmented marks (stains) on the skin, these areas signifying merely that inflammatory lesions had been present, but the active quality (redness) had entirely disappeared; following severe inflammation, it may require many weeks for the stain to disappear.

War dermatology consists chiefly of animal parasites and their complications. Exposure, skin trauma, and vegetable organisms are responsible for a large toll.

*Conclusions.* All diseases of the skin, exclusive of uncomplicated cases of scabies and pediculosis, should be treated in areas where dermatological advice is available.

All skin cases requiring prolonged treatment should be isolated under the care of a dermatologist.

A dermatologist subject to call is indispensable for the diagnosis of cases of skin diseases of unusual type or rare conditions.

